# Inflammatory parameters and color alterations of dental bleaching in patients wearing fixed orthodontic appliance: a randomized clinical trial

**DOI:** 10.1186/s12903-023-03301-7

**Published:** 2023-08-28

**Authors:** Edson Gustavo Pereira Barbosa, Suellen Nogueira Linares Lima, Júlio de Araújo Gurgel, Elizabeth Soares Fernandes, Sebastião Marinho Pinheiro Neto, Rudys Rodolfo de Jesus Tavarez, Karine Letícia da Silva, Alessandro D. Loguercio, Célia Regina Maio Pinzan-Vercelino

**Affiliations:** 1 CEUMA University, São Luís, Brazil; 2 State University of São Paulo, Marília, SP Brazil; 3 Pequeno Príncipe Faculty and Research Institute Pelé Pequeno Príncipe, Curitiba, PR Brazil; 4 Department of Restorative Dentistry, Ponta Grossa State University, Rua Carlos Cavalcanti, 4748, Bloco M, Sala 64A – Uvaranas, Ponta Grossa, 84030- 900 Paraná Brazil; 5 Department of Orthodontics, University Center Ingá, Maringá, PR Brazil

**Keywords:** Orthodontic brackets, Orthodontic appliances, Fixed, Tooth bleaching, Gingival crevicular fluid, Inflammation, Colour

## Abstract

**Background:**

Many orthodontic patients request dental bleaching during orthodontic treatment to achieve a faster aesthetic resolution, however, no attention has been paid to the inflammatory processes that can occur when both therapies are indicated together. So, this clinical trial evaluated the inflammatory parameters and color alterations associated with dental bleaching in patients wearing a fixed orthodontic appliance.

**Methods:**

Thirty individuals aged between 18 and 40 years were equally and randomly allocated into three groups: FOA (fixed orthodontic appliance), BLE (dental bleaching), and FOA + BLE (fixed orthodontic appliance + dental bleaching). The orthodontic appliances and the bleaching procedures were performed in the maxillary premolars and molars. For dental bleaching a 35% hydrogen peroxide was used. The gingival crevicular fluid (GCF) and nitric oxide (NO^-^) levels were evaluated at different time-points. Color evaluation was performed using an Easyshade spectrophotometer at baseline (FOA, FOA + BLE, BLE), one month after (FOA + BLE) and 21 days after appliance removing (FOA + BLE and FOA groups), in each tooth bleached. The ANOVA and Tukey’s tests, with a significance level of 5%, were used for statistical analysis.

**Results:**

The GCF volume in the FOA + BLE and FOA groups significantly increased at the time points evaluated (*p* < 0.001); however, this did not occur in the BLE group (*p* > 0.05). On the other hand, NO^-^ levels significantly decreased during dental bleaching with or without fixed orthodontic appliances (FOA + BLE and BLE groups; *p* < 0.05), while no significant changes were observed in the FOA group (*p* > 0.05). Significant changes in color were observed in the FOA + BLE and BLE groups compared to in the FOA group (*p* < 0.01). However, the presence of fixed orthodontic appliance (FOA + BLE) negatively affected the bleaching efficacy compared to BLE group (*p* < 0.01).

**Conclusions:**

Dental bleaching did not increase the inflammatory parameters in patients wearing fixed orthodontic appliance. However, in the presence of orthodontic appliances, the bleaching efficacy was lower than that of bleaching teeth without orthodontic appliances.

**Trial registration:**

RBR-3sqsh8 (first trial registration: 09/07/2018).

## Introduction

Aesthetics are one of the main demands of patients seeking dental treatment. Therefore, among the most common complaints are the changes in dental color, shape, and teeth alignment [1]. This is mainly due to the growing awareness that aesthetically aligned and white teeth are standards of an ideal smile [2]. Therefore, dental bleaching and orthodontics are two of the most commonly used procedures to improve the aesthetics of smile [3–5].

According to Slack et al. [6], approximately 90% of orthodontists in the United States reported that their patients requested dental bleaching at some point during their orthodontic treatment, which highlights the need for orthodontists to stay updated in dental bleaching research. Currently, mostly orthodontic patients still request dental bleaching procedure during orthodontic treatment [5, 7], and to achieve a faster aesthetic resolution, a whitening effect and a better position of teeth are simultaneously obtained. Despite the use of these procedures, there is usually no indication of using both the procedures simultaneously [5, 7, 8]. After dental alignment, which usually occurs in the first stage of orthodontic treatment, several patients request dental bleaching because color dissatisfaction increases when the crowding is resolved [1].

However, similar clinical trials have demonstrated the effectiveness of at-home bleaching performed on teeth with orthodontic brackets [3, 4, 6–9]. These studies suggest that the low molecular weight of hydrogen peroxide can allow a polydirectional flow inside the enamel and dentin, allowing the removal superficial and deep stains by the oxireduction reaction, thus bleaching the dental structure under the orthodontic bracket area [3, 4].

However, no attention has been paid to the inflammatory processes that can occur when both therapies are indicated together. For instance, it is well known that the fixed orthodontic appliance (FOA) is composed of accessories and wires that can facilitate the accumulation of biofilm and cause inflammation of periodontal tissues [10–13]. However, in the early stages of gingivitis, the periodontium can be observed with a change in the volume of gingival crevicular fluid (GCF) and a gradual release of inflammatory mediators [10, 11, 14]. The GCF is one of the indicators of inflammatory activity [15–17], and the concentrations of nitrites and nitrates produced by the immunological response during the pathogen-host interaction can be evaluated, which plays an important role in understanding the etiopathogenesis of periodontal disease associated with orthodontic appliances and bleaching materials [18].

Regarding the adverse effects that occur during bleaching procedures, the concerns mainly related to tooth sensitivity and gingival irritation. However, the evidence gathered to date regarding the inflammatory aspects of dental bleaching remains controversial. Despite not detecting any inflammatory changes post dental bleaching, recent randomized clinical trials [18, 19] reported a moderate and transient inflammation immediately after the bleaching procedure, which returned to a normal level 24 h after the application of bleaching gel. On the other hand, Colares et al. [15] and Bersezio et al. [20] demonstrated that dental bleaching agents can induce crevicular inflammation and damage, and an inflammatory response largely associated with nitric oxide synthesis and leukocyte activation [15, 20]. This inflammatory process persists for 6 months [21].

Therefore, considering that previous studies have demonstrated changes in inflammatory activity using FOAs [10–13] as well as using dental bleaching [15, 20, 21], it is important to evaluate the inflammatory process when both procedures are applied together.

Thus, the primary objective of this clinical trial was to investigate the alterations in inflammatory activity in patients undergoing both dental bleaching and orthodontic treatment in comparison with that in patients who underwent either dental bleaching or orthodontic treatment. In addition, the efficacy of dental bleaching in teeth with and without orthodontic appliances was also evaluated. The null hypotheses tested were: (1) there was no difference in inflammatory activity when dental bleaching or orthodontic treatment was used alone; and (2) dental bleaching performed during the orthodontic treatment would not result in different degrees of color changes when compared to dental bleaching performed alone.

## Methods

### Ethics approval and protocol registration

This clinical trial investigation was approved by the local Research Ethics Committee (protocol # 2.647.386) of CEUMA University (São Luís, MA, Brazil), and written informed consent was obtained from all participants. Participants were informed of the nature and objectives of the present study. The study was registered in the Clinical Trials Registry (RBR-3sqsh8; first trial registration: 09/07/2018). The protocol established by the Consolidated Standards of Reporting Trials (CONSORT) [22] statement was followed.

### Study design and recruitment

This was a parallel randomized clinical trial with an equal allocation rate for one of the three protocols evaluated. The study was conducted in the clinical setting of the School of Dentistry of the CEUMA University from May 2018 to November 2018. Once the criteria were established, 30 volunteers were selected for the present study. Recruitment was performed by posting advertisements at the local university.

### Inclusion and exclusion criteria

The patients included in this clinical trial were 18–40 years of age, had good general and oral health, and required orthodontic treatment. Each participant was required to have premolar teeth that were color shade A3 or darker, which as determined by comparison with a value-oriented shade guide (Vita Classical; VITA Zahnfabrik, Bad Säckingen, Germany), and caries-free with no restorations on any surface. The participants needed to have the presence of all permanent teeth up to the erupted first molars and normal alignment and leveling of the canines, premolars and molars. In addition, only participants with no signs of periodontal disease, or systemic disorders capable of interfering with the periodontal condition (e.g., diabetes), and those who did not use antimicrobial or antibiotics solutions in the last 3 months before the study began were included.

Participants were excluded if they had poor oral hygiene, were pregnant or lactating women, those who had undergone dental bleaching treatment, and smokers. Participants with visible cracks, gingival recession, carious or non-carious cervical lesions, spontaneous tooth sensitivity, severe internal discoloration, or bruxism were excluded. Participants taking medications like analgesic or anti-inflammatory drugs were also excluded.

All participants were informed of the nature and objectives of the study and the informed consent was obtained from all subjects and/or their legal guardian(s).

### Sample size calculation

The primary outcome of this study was inflammatory activity. However, as the three groups were not compared, two different sample size calculations were performed. Regarding the comparison between FOA + BLE vs. FOA, the sample size calculation was based on data published by van Gastel et al. [13], using the volume of gingival fluid as a reference variable. The parameters used were a standard deviation of 0.25 µl, and a minimum difference of 0.38 µl between the means (G*Power software, version 3.1.3; Franz Faul, University of Kiel, Kiel, Germany), a confidence level of 95% and a power of 80%. Therefore, the minimum number of participants was determined to be 10 per group.

Regarding the comparison between FOA + BLE vs. BLE, the sample size calculation was based on data published by Lima et al. [19], using the volume of gingival fluid as a reference variable. The parameters used were a standard deviation of 0.15 µl, and a minimum difference of 0.23 µl between the means (G*Power software, version 3.1.3; Franz Faul, University of Kiel, Kiel, Germany), a confidence level of 95% and a power of 80%. Therefore, the minimum number of participants was determined to be 10 per group.

### Random sequence generation and allocation concealment

The randomization process was performed by a computer-generated lists prepared by a third person not involved in the research protocol, using the website www.sealedenvelope.com. Randomization was carried out in blocks to obtain the same proportion of participants in each group. Details of the allocated group were recorded on cards posted in opaque and sealed envelopes sequentially numbered. These envelopes were opened only in the first appointment to prevent the disclosure of the randomization scheme. Neither the participant nor the operator knew the group allocation before the card opening.

### Study intervention

One week before initiating the treatment, all participants received dental prophylaxis with rubber cups and pumice. In this session, a matrix was created for each participant using condensation silicone (Coltoflax and Perfil Cub; Vigodent, Rio de Janeiro, RJ, Brazil) to standardize the area of color measurement. In the matrix, a circular window was performed in the buccal surface of each tooth. It was configured using a circular metal cutting device measuring 6 mm in diameter (Biopsy Punch (Miltex, York, Pensilvania, USA), corresponding to the diameter of the spectrophotometer device (Easyshade Advance 4.0; Vident, Brea, CA, USA) [3, 4, 19]. The 30 participants were equally allocated into three groups: only FOA, only dental bleaching (BLE), and FOA + BLE.

### Only fixed orthodontic appliance (FOA) group

The volunteers in the FOA group had ceramic brackets and metal tubes (straight wire, Roth prescription, slot 0.022 × 0.030-inch) passively bonded to their first premolars, second premolars, and first molars on both the maxillary quadrants with Orthocem self-adhesive resin cement (FGM Prod. Odontol. Ltda., Joinville, SC, Brazil) according to the manufacturer’s instructions. With the matrix installed on the teeth, the brackets were positioned at the circular opening located in the middle third of the buccal surface of the teeth. Prior to photopolymerization, the matrix was carefully removed, and the brackets were adjusted with the slots aligned using a rectangular steel archwire (0.018 × 0.025-inches) as a guide, in order to not cause tooth movement. A single operator, who was a specialist in orthodontics, performed all the bonding procedures. The 0.014-inch diameter round cross-section nickel-titanium wires were tied into the slot by using aesthetic ligatures.

In this group, accessories were removed after 42 days of treatment. Bracket-removing pliers (GAC, Dentsply, New York, USA) were used to debond the accessories. The remaining resin was removed with a 9-bladed tungsten carbide bur (CB27; Orthometric, Haidian, Beijing, China) in a hand piece at a low speed. Final polishing was performed with gloss paste (Poligloss, TDV, Pomerode, SC, Brazil) using a rubber cup at a low speed for 20 s. Subsequently, the surfaces were washed with water for 20 s and dried for 10 s.

### Only dental bleaching (BLE) group

In this group, the gingival tissue, lips and cheeks of the teeth to be bleached were isolated using a light-polymerized resin dam (Top Dam; FGM Prod. Odontol. Ltda., Joinville, SC, Brazil). Subsequently, 35% hydrogen peroxide was used for in-office dental procedures (Whiteness HP Maxx, FGM Prod. Odontol. Ltda., Joinville, SC, Brazil). The gel was applied for 15 min in triplicate. Two dental bleaching sessions were performed with a 1-week interval between sessions.

### Fixed orthodontic appliance + dental bleaching (FOA + BLE) group

The same procedure was performed in this group as in the FOA and BLE groups. However, the bleaching procedure, as described in BLE group, was performed with the brackets in position. For this purpose, the orthodontic wire and aesthetic ligatures were removed before dental bleaching and reinstalled after the procedure. In this group, the FOA was removed 7 days after the second bleaching session, corresponding to 42 days of treatment.

As in the BLE group, 35% hydrogen peroxide gel was used (Whiteness HP Maxx, FGM Prod. Odontol. Ltda., Joinville, SC, Brazil) for 15 min in triplicate, and two dental bleaching sessions were performed with a 1-week interval. In the FOA + BLE group, gel was applied around the orthodontic bracket until it completely covered the tooth to be bleached, as previous described by Gomes et al. [8].

For all groups, participants were instructed to brush their teeth regularly using toothpaste without desensitizing or bleaching agents during the entire investigation period. The participants were also instructed to use dental floss on interproximal surfaces. To ensure ethical considerations, individuals assigned to the FOA group who desired teeth bleaching underwent the bleaching procedure after the final data collection. Similarly, individuals assigned to the BLE group who desired fixed orthodontic appliances underwent the procedure after the final data collection. Following the conclusion of the research study, all individuals in the FOA groups (FOA and BLE + FOA) received treatment with the fixed orthodontic appliance according to the predefined treatment plan for each clinical case.

### Collection of gingival crevicular fluid (GCF)

To collect the GCF, the areas were isolated with cotton rolls and the teeth were gently dried with air for 10 s [19]. The site chosen for sample collection were the mesiobuccal and distobuccal areas of the four maxillary premolars of each participant. This was done using a standard paper strip (Perio-paper; IDE Interstate, Amityville, NY, USA) which was inserted into the sulcus to a depth of 1–2 mm for 15 s [14]. After removal, each strip was immediately inserted into a calibrated moisture meter (Periotron 8010; Oraflow Inc., Smithtown, NY, USA) to determine the GCF volume. Strips contaminated with blood were excluded from the sample group, and the measurement was repeated at another site. After collecting the gingival fluid, the strips were immediately placed in sterile Eppendorf tubes, and the GCF samples were stored at -80 °C until subsequent analysis. The measurements were performed at the time points [19, 23] described in Table 1 for each group.

**Table 1:** Time-points of gingival crevicular fluid collection

Time-Points	Group FOA + BLE		Group FOA		Group BLE
**T0**	Before FOA installation		Before FOA installation		Before first bleaching session
**T1**	28 days after FOA installation/ immediately before first bleaching session		28 days after FOA installation		
**T2**	24 h after first bleaching session				24 h after first bleaching session
**T3**	Before second bleaching session				Before second bleaching session
**T4**	24 h after second bleaching session				24 h after second bleaching session
**T5**	Before FOA removal		Before FOA removal		
**T6**	21 days after FOA removal/ 28 days after second bleaching session		21 days after FOA removal		21 days after second bleaching session
T0 = T1 for group BLE					

### Nitric oxide levels

Strips containing GCF were incubated individually with 160 µl of phosphate-buffered saline for 10 min and vortexed every 2 min of incubation. The samples were then centrifuged at 3000 rpm for 5 min. The resulting supernatants were used for assays.

To determine the NO2^-^/NO3^-^ content (as NO- end products) in each of the samples the Griess assay was used an indicator of NO production, as described previously [26]. NO_3_^-^ was reduced to nitrite (NO_2_^-^) by incubating 80 µL of sample with 20 µL of 1 U/ml nitrate reductase and 10 µL of 1 mM NADPH during 30 min at 37 °C in a 96-well plate. Then, 100 µl Griess reagent (5% v/v H_3_PO_4_ containing 1% w/v sulfanilic acid and 0.1% w/v N-1-napthylethylenediamine) was added and incubated for 15 min at 37 °C. The absorbance at 550 nm was immediately measured using a spectrophotometer (MB-580; Heales, Shenzhen, China). After subtraction of the background readings, the absorbance of each of the samples was compared with that obtained from a sodium nitrite (0-100 µM) standard curve and expressed as NO^-^ concentrations (µM).

### Color evaluation

Color measurements were made using a VITA Easyshade spectrophotometer (Easyshade; Vident, Brea, CA, USA). Color evaluation was performed by one operator using the matrix previously described to standardize the placement of the spectrophotometer device in the same place during consecutive color evaluations [3, 4, 19].

The color was determined using the parameters of the Easyshade device, which indicated the following values: L*, a* and b*, where L* represents the value from 0 (black) to 100 (white); a* and b* represent the shade, where a* is the measurement along the red-green axis and b* is the measurement along the yellow-blue axis. The color comparison before and after each period of treatment (Table 1) was determined by the difference between the two shades (ΔE), which was calculated using the following formula: Δ*E* = [(ΔL*)^2^ + (Δa*)^2^ + (Δb*)^2^]^1/2^ [24]. In addition, it was used the Whiteness index for Dentistry according to the following formula: WI_D_ = 0.511 L* − 2.324a* − 1.100b* [25]. Three readings were obtained, and the values were averaged for statistical purposes. The color measurements were objectively recorded at baseline and one month after starting the bleaching session in the FOA + BLE and BLE groups, and 21 days after removing the appliance in the FOA + BLE and FOA groups (Table 1).

### Statistical analysis

Regarding the GCF and NO^-^ level data, all measurements performed in each participant were averaged for statistical purposes. All measurements were submitted to the Kolmogorov–Smirnov test to evaluate the data distribution. For inflammatory activity parameters, two comparisons were performed: (1) FOA vs. FOA + BLE groups at T0, T1, T5, and T6, and (2) FOA + BLE vs. BLE groups at T0, T2, T3, T4, and T6. For intergroup comparison at each time point, the Student’s t-test for independent variables was applied, and for intragroup comparison, the one-way ANOVA for dependent variables and Tukey’s test as a post-hoc comparison were applied. For color evaluation, all groups were subjected to one-way ANOVA for independent variables, as well as Tukey’s post-hoc test for each of the parameters evaluated (ΔL*, Δa*, Δb*, ΔE_ab_, and ΔWI_D_). The level of significance adopted was 5%. SPSS software (IBM, Chicago, IL, USA) was used for statistical analysis.

## Results

### Characteristics of included participants

**Fig. 1:**
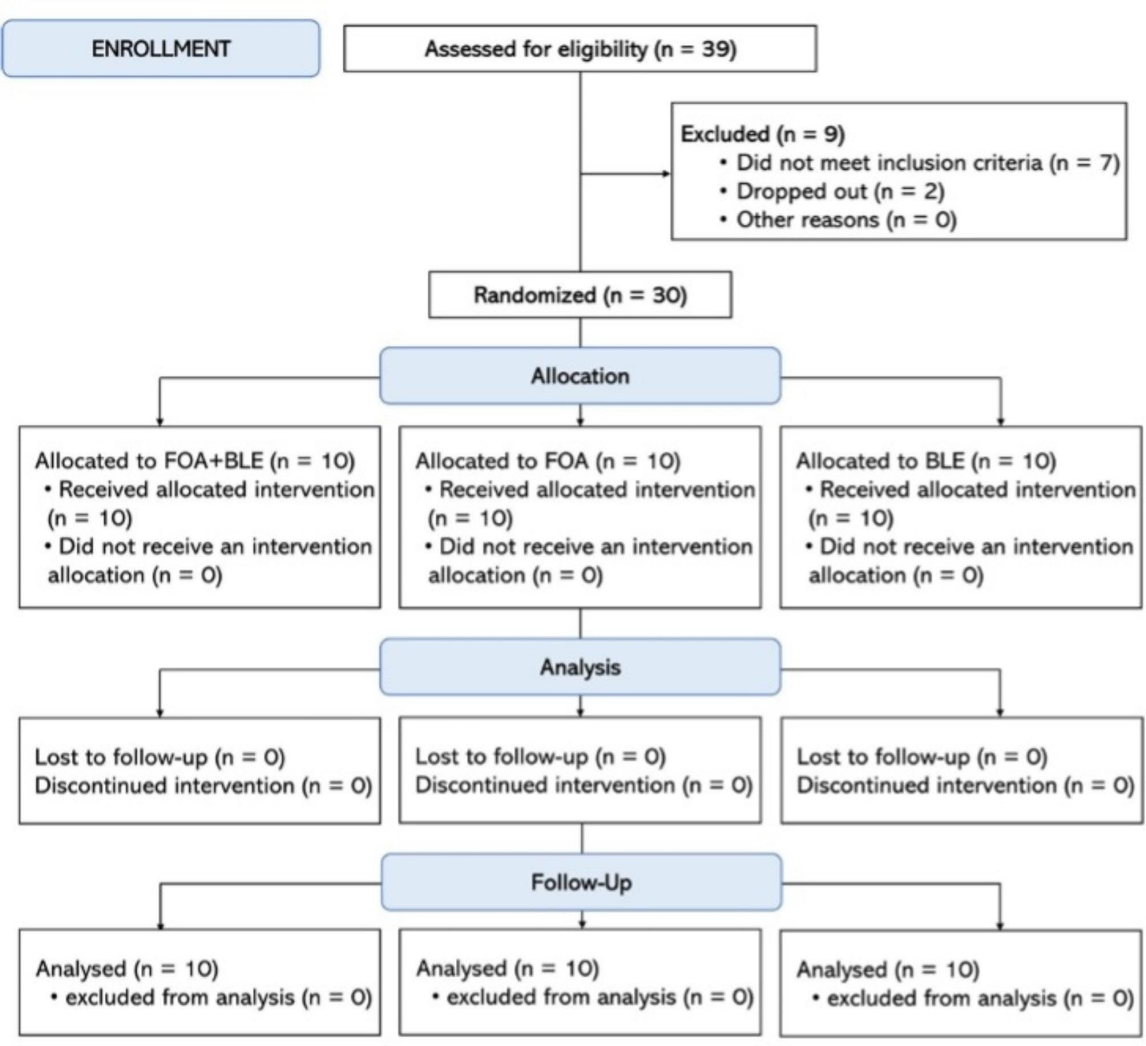
Flow diagram of the clinical trial

Thirty-nine participants were examined, and nine were excluded based on the inclusion and exclusion criteria (Fig. 1). The baseline color parameters of the participants and the age distribution were similar, as described in Table 2.

**Table 2:** Characteristics of participants: initial age (years) and baseline color parameters (L*, a* and b* values)

	FOA + BLE Group	FOA Group	BLE Group	*p*-value (One-way ANOVA)
Characteristics	Mean ± SD	Mean ± SD	Mean ± SD	
Age	22.8 ± 3.27	23.0 ± 2.4	20.7 ± 2.4	0.10
L*	80.5 ± 3.39	79.6 ± 5.0	79.7 ± 4.8	0.67
a*	0.74 ± 0.98	0.37 ± 1.5	0.33 ± 0.8	0.29
b*	23.6 ± 3.16	23.4 ± 4.1	22.9 ± 4.9	0.85

### Adherence to the protocol

The adherence to the protocol was 100%. Figure 1 shows the participant flow diagram for the different phases of the study design.

### Gingival crevicular fluid (GCF) volume

The results showed a statistically significant increase in GCF volume in the FOA + BLE group compared with that in the FOA group at T5 (Table 3; *p* < 0.01). Intragroup comparisons demonstrated that in both the FOA + BLE and FOA groups, a statistically significant increase in GCF volume occurred between T0 and T1 (*p* < 0.01), and these higher levels were maintained during all the periods evaluated. When the FOA + BLE group was compared with the BLE group, a statistically significant increase in GCF volume for the former was observed at T2 and T3 (Table 3; *p* < 0.01). Intragroup comparisons demonstrated no significant increase in GCF volume in the BLE group (*p* > 0.05).

**Table 3:** Mean values, standard deviations, and gingival fluid (µL) comparison between groups (horizontal) and time-points (vertical)

Time-points	FOA + BLE	FOA	*p*-value*	Time-points	FOA + BLE	BLE	*p*-value*
	Mean ± SD	Mean ± SD			Mean ± SD	Mean ± SD	
T0	0.20 ± 0.14^aA^	0.18 ± 0.15^aA^	0.346	T0	0.20 ± 0.14^aA^	0.21 ± 0.15^aA^	0.546
T1	0.61 ± 0.2^bA^	0.40 ± 0.15^bA^	0.346	T2	0.64 ± 0.13^bA^	0.38 ± 0.21^aB^	0.004
T5	0.54 ± 0.17^bA^	0.36 ± 0.13^bB^	0.01	T3	0.58 ± 0.17^bA^	0.35 ± 0.18^aB^	0.009
T6	0.42 ± 0.24^bA^	0.47 ± 0.16^bA^	0.547	T4	0.64 ± 0.21^bA^	0.41 ± 0.21^aA^	0.184
				T6	0.42 ± 0.24^bA^	0.32 ± 0.22^aA^	0.547
*p*-value**	< 0.001	< 0.001		*p*-value**	< 0.001	0.123	

(*) Both groups at each time point were evaluated using the Student’s t-test for independent groups. Similar capital letters indicate no significant difference. (**) Each group at different times was evaluated by One-way ANOVA for dependent groups and Tukey’s test. Similar lowercase letters indicate no significant difference (*p* > 0.05)

### NO-concentrations

**Fig. 2:**
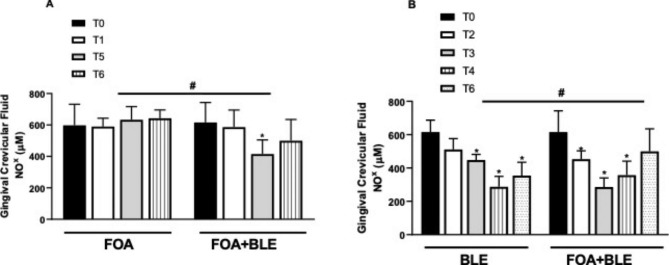
Analysis of gingival crevicular fluid (GCF) volume of the different groups

All the data for NO concentrations are depicted in Fig. 2. There were significant differences between the FOA + BLE vs. FOA, as well as BLE vs. FOA + BLE groups (*p* < 0.05). The intragroup comparisons demonstrated that while the GCF NO- concentrations were similar during the time points evaluated for the FOA group (*p* > 0.05), for the BLE group, there was a statistically significant reduction in GCF NO- concentrations at T3, T4, and T6 in relation to the baseline values (*p* < 0.05). In addition, the FOA + BLE group showed a statistically significant reduction in GCF NO- concentrations at T2, T3, T4, and T5 compared with that at baseline concentrations.

### Color evaluation

When bleaching was applied as in FOA + BLE and BLE groups, a significant improvement in the color parameters was observed when compared to that in the FOA group (Table 4). However, different results were observed when both the groups (FOA + BLE and BLE) were compared to FOA (Table 4; *p* < 0.001). In the BLE group, all the color parameters were significantly different from those in the FOA group (Table 4; *p* < 0.001). However, no significant differences were observed when ΔL* and ΔE_ab_ values of FOA + BLE and FOA groups were compared (Table 4; *p* > 0.05). When the BLE and FOA + BLE groups were compared, a significant difference was only observed in the ΔWI_D_ color parameter, favoring the BLE group (Table 4; *p* < 0.001).

**Table 4:** Mean and standard deviations of different color parameters evaluated*

	FOA + BLE		FOA		BLE	*p*-value
	Mean ± SD		Mean ± SD		Mean ± SD	
ΔL*	0.70 ± 3.5^ab^		-1.25 ± 3.5^b^		2.24 ± 5.8^a^	0.003
Δa*	− 1.80 ± 1.6^a^		− 0.25 ± 1.1^b^		− 1.83 ± 1.3^a^	< 0.001
Δb*	− 5.77 ± 5.5^a^		− 0.95 ± 4.1^b^		− 6.67 ± 4.3^a^	< 0.001
**ΔE**_**ab**_	7.46 ± 3.1 ^ab^		4.75 ± 2.4^b^		9.37 ± 1.9^a^	0.001
**Δ**WI_**D**_	5.63 ± 9.3^b^		− 3.44 ± 4.8^c^		9.79 ± 6.2^a^	< 0.001

(*) All groups for each color parameter were evaluated using One-way ANOVA and Tukey’s test. Similar lowercase letters indicate no significant difference (*p* > 0.05)

## Discussion

The results found on this study showed that bleaching during orthodontic treatment does not appear to be related to an increase in GCF volume. Since GCF collection is a simple and non-invasive procedure, it has been used as a potential indicator of an ongoing inflammation and a useful tool for monitoring post-dental procedures [27]. However, it has only recently been used to evaluate pro-inflammatory alterations associated with dental bleaching [28]. A closer view of these articles showed that no significant changes in GCF volume were observed when in-office dental bleaching was applied, suggesting that this procedure does not cause inflammation at the GCF level [15, 19, 28].

However, on the other hand, in the FOA group there was a significant increase in the GCF volume, mainly after 28 days of treatment (T1) as expected [28], with no significant increase when associated with dental bleaching (FOA + BLE). As in orthodontics no tooth movement can be achieved without inflammation, the inflammatory process is an event natural towards solving functional and/or aesthetic problems. However, orthodontically induced inflammation and inflammatory mediators found in this process are transitory [29, 30]. A closer view of the results showed that the GCF volume tended to decrease, mainly 21 days after orthodontic appliance removal, in agreement with previous studies [30, 31].

Interestingly, despite increasing the GCF volume, the GCF NO- concentration did not change in the FOA group. This means that the increase in GCF volume observed in this study could not be correlated with inflammation after orthodontic movements; however, it was correlated with a stimulus inducing fluid production.

In contrast, a significant reduction in the GCF NO- concentrations was detected in the BLE and FOA + BLE groups. In the case of the FOA + BLE group, the bleaching procedure was performed 28 days after the installation of the FOA, because, according to previous studies, inflammatory activity had already been observed [10, 11]. These data indicate that the application of bleaching was the most important factor in the reduction of GCF NO- concentrations.

According to the study by Colares et al. [15], dental bleaching with high hydrogen peroxide concentrations caused damage to NO-producing cells (neurons and endothelial cells) by reducing the NO- levels during the procedure. The production of nitric oxide [10] occurs in parallel as part of nitroactive stress, due to the action of the nitric oxide synthase enzyme, resulting from the union of arginine with O_2_. Once formed, NO can react with HO- and generate peroxynitrite (ONOO-), which in turn causes tissue damage ecause it promotes changes in DNA and cellular proteins, thereby causing apoptosis of these cells and often loss of tissue function.

Dental bleaching agents have been suggested to cause tissue damage, a response that is associated with the reduction of GCF NO- levels from 1 to 7 days following dental bleaching [15]. In this study, NO- was increased in samples of control participants (T1) post-bleaching, indicating that the ongoing local inflammation was in course and was first detected in the GCF and later in (T2) gingival fluid samples. However, in contrast to the findings of Colares et al. [15], NO- levels decreased before the 2nd session up to 28 days after bleaching and did not return to baseline levels in the final time, mainly in the BLE group.

Despite the GCF and NO- concentrations are consider the most adequate method to detect inflammation in patients submitted to in-office bleaching [15, 19, 28]. It is worth to mention that several other inflammatory markers can be selected to evaluate the effect of bleaching agents. However, previous studies showed that in-office bleaching as applied in the present study did not increase of several cytokines evaluated [15, 19, 28].

Unfortunately, several methodological differences between the present and previous studies [15] must be considered when different studies are compared. Differences between pH and the presence of additional substances in bleaching gels could affect the speed of the release of hydrogen peroxide and, consequently, their potential for cellular damage [32, 33].

Regarding color change, the results of this study showed that the application of hydrogen peroxide at higher concentrations using in-office bleaching demonstrated significant tooth color enhancement during orthodontic treatment when compared with that of the baseline. Although this was in agreement with previous clinical studies [3, 4], this was the first randomized clinical study evaluating in-office bleaching associated with orthodontic appliances, as only case reports regarding these associations have been published [4, 6–9]. In addition, previous studies must be considered observational [3, 4], since only one group (bleaching associated with orthodontic treatment) was evaluated.

It can be observed that the bleaching agent has the ability to bleach teeth uniformly, even when not in direct contact with the buccal surface. This is because hydrogen peroxide diffusion occurs in multiple directions throughout the tooth structure, allowing for homogeneous bleaching [3, 4]. This phenomenon has been supported by previous clinical studies, which demonstrated that no noticeable color difference was observed after the removal of fixed orthodontic appliances when comparing the area under the appliances and the remaining surface surrounding them [3, 4].

This can be explained by the permeability of the tooth structure and the lower molecular weight of hydrogen peroxide. Both factors allow that low molecular weight molecules of hydrogen peroxide to easily cross through the enamel surface to dentin by the formation of free radicals that interact with the pigmented organic molecules, destabilizing and bleaching them, and thus producing the whitening effect [34, 35]. When in contact with dentin, hydrogen peroxide can act in a multidirectional manner, reaching even under and around the brackets and adhesives of the cementation resins of orthodontic appliances [36].

It is worth mentioning that both color measurements were superior to 50:50 perceptibility (PT) and 50:50 acceptability threshold (AT). For ΔE, the 50:50 PT value was reported to be 1.2, and the 50:50 AT value was reported to be 2.7 [37], whereas for ΔWI_D_ the values were 0.7 and 2.6 for PT and AT, respectively [37]. This means that the bleaching observed was superior to the minimal color difference that human eyes can distinguish (PT), and that these differences were acceptable for most people (AT). Nevertheless, all color measurements were performed after the removal of the fixed orthodontic appliance. It is important to note that in routine clinical care, evaluating bleaching while undergoing treatment with a fixed orthodontic appliance can have a negative impact on PT and AT by patients.

However, when the BLE group was compared with the FOA + BLE group, some differences were observed when both parameters were compared (ΔE_ab_ and ΔWI_D_), as previously reported by in vitro studies [38, 39]. Despite the availability of new formulas, the CIEL_ab_ formula (ΔE_ab_) was the most extensively used color parameter in the literature, and this was the main reason why it was presented in this study. Unfortunately, ΔE_ab_ cannot give the magnitude of color change, and this was the main reason for using the WI_D_, which indicates the degree of whitening towards the lighter end of the scale [24, 36].

Therefore, it is important to compare both the groups using ΔWI_D_. There was a significantly higher whitening efficacy for the BLE group than for the FOA + BLE group, since these differences of means between groups reached the PT and AT thresholds, being therefore clinically important. Despite the difference in the whitening efficacy observed, the technique in orthodontic patients should still be considered clinically important, since many patients request dental bleaching during orthodontic treatment, aiming of obtaining white and well-aligned teeth as soon as possible [5, 7, 8], since after the removal of orthodontic brackets, dissatisfaction with tooth coloring seems to increase [1].

Considering that this was the first randomized clinical study that showed that the use of orthodontic appliances disturbs the bleaching process to some degree, it seems to be an important conclusion. However, the bleaching efficacy can be potentially improved with one additional session of in-office bleaching [40] or by associating the present treatment with at-home bleaching [41, 42, 43] Future clinical studies evaluating this hypothesis should be conducted.

Finally, it is worth mentioning that despite the present study have evaluated all hypothesis in posterior teeth, which is not consider teeth in an aesthetic area, it will be expected the same events, in terms of bleaching effect or due to underwent fixed orthodontic appliance in the anterior teeth. Also, as in-office bleaching using high-concentrated hydrogen peroxide in forbidden in several parts of the World, mainly in the European community, this negatively impact the external validity of the present study.

Actually, dental bleaching can act as a motivating factor, avoiding patient withdrawal or interruption of treatment. However, as in the present study, the use of orthodontic appliance was the most important factor influencing the inflammatory activity in periodontal tissue. It demonstrated that participants undergoing orthodontic treatment who choose dental bleaching need to have good periodontal health to perform this procedure. If we are taking in account the increase of use of aligners instead fixed appliance, and the use of the same aligners as bleaching tray, this could be other interesting suggestion to associate orthodontic aligners with at-home bleaching [44, 45].

## Conclusions

When in-office dental bleaching using 35% hydrogen peroxide was performed in individuals wearing a FOA, an increase in nitric oxide levels was attributed to the bleaching agent, and an increase in the GCF was attributed to the orthodontic treatment. The presence of fixed orthodontic appliances negatively affects bleaching.

## Data Availability

Data and materials are available when request for the authors. All methods were carried out in accordance with relevant guidelines and regulations. Alessandro D. Loguercio should be contacted to request the data from this study.

## Abbreviations

AT: Acceptability
BLE: Dental bleaching
CONSORT: Consolidated Standards of Reporting Trials
FOA: Fixed orthodontic appliance
GCF: Gingival crevicular fluid
NO: Nitric oxide
PT: Perceptibility

## References

[1] Lawson J, Warren JJ, Levy SM, Broffitt B, Bishara SE (2008). Relative esthetic importance of orthodontic and color abnormalities. Angle Orthod.

[2] Alani A, Kelleher M, Hemmings K, Saunders M, Hunter M, Barclay S, Ashley M, Djemal S, Bishop K, Darbar U (2015). Balancing the risks and benefits associated with cosmetic dentistry - a joint statement by UK specialist dental societies. Br Dent J.

[3] Jadad E, Montoya J, Arana G, Gordillo LA, Palo RM, Loguercio AD (2011). Spectrophotometric evaluation of color alterations with a new dental bleaching product in patients wearing orthodontic appliances. Am J Orthod Dentofacial Orthop.

[4] Montenegro-Arana A, Arana-Gordillo LA, Farana D, Davila-Sanchez A, Jadad E, Coelho U, Gomes O, Loguercio AD (2016). Randomized double-blind clinical trial of Bleaching Products in patients wearing Orthodontic Devices. Oper Dent.

[5] Sulieman MA (2008). An overview of tooth-bleaching techniques: chemistry, safety and efficacy. Periodontol 2000.

[6] Slack ME, Swift EJ, Rossouw PE, Phillips C (2013). Tooth whitening in the orthodontic practice: a survey of orthodontists. Am J Orthod Dentofacial Orthop.

[7] Thickett E, Cobourne MT (2009). New developments in tooth whitening. The current status of external bleaching in orthodontics. J Orthod.

[8] Gomes MN, Dutra H, Morais A, Sgura R, Devito-Moraes AG (2017). In-Office bleaching during Orthodontic Treatment. J Esthet Restor Dent.

[9] Sundfeld RH, Machado LS, de Oliveira FG, Santos EA, Lugato IC, Sundfeld Neto D (2012). Conservative reconstruction of the smile by orthodontic, bleaching, and restorative procedures. Eur J Dent.

[10] Bergamo AZ, Nelson-Filho P, Romano FL, da Silva RA, Saraiva MC, da Silva LA, Matsumoto MA (2016). Gingival crevicular fluid volume and periodontal parameters alterations after use of conventional and self-ligating brackets. J Orthod.

[11] Bergamo AZN, Nelson-Filho P, do Nascimento C, Casarin RCV, Casati MZ, Andrucioli MCD, Kuchler ÉC, Longo DL, da Silva LAB, Matsumoto MAN (2018). Cytokine profile changes in gingival crevicular fluid after placement different brackets types. Arch Oral Biol.

[12] Jurela A, Repic D, Pejda S, Juric H, Vidakovic R, Matic I, Bosnjak A (2013). The effect of two different bracket types on the salivary levels of S mutans and S sobrinus in the early phase of orthodontic treatment. Angle Orthod.

[13] van Gastel J, Teughels W, Quirynen M, Struyf S, Van Damme J, Coucke W, Carels C (2011). Longitudinal changes in gingival crevicular fluid after placement of fixed orthodontic appliances. Am J Orthod Dentofacial Orthop.

[14] Gupta S, Chhina S, Arora SA (2018). A systematic review of biomarkers of gingival crevicular fluid: their predictive role in diagnosis of periodontal disease status. JOBCR.

[15] Colares VLP, Lima SNL, Sousa NCF, Araújo MC, Pereira DMS, Mendes SJF, Teixeira SA, Monteiro CA, Bandeca MC, Siqueira WL (2019). Hydrogen peroxide-based products alter inflammatory and tissue damage-related proteins in the gingival crevicular fluid of healthy volunteers: a randomized trial. Sci Rep.

[16] De Lima AJ, Van Dyke TE (2003). Origin and function of the cellular components in gingival crevice fluid. Periodontol 2000.

[17] Wassall RR, Preshaw PM (2016). Clinical and technical considerations in the analysis of gingival crevicular fluid. Periodontol 2000.

[18] Topcu Ali O, Akalin FA, Sahbazoglu KB, Yamalik N, Kilinc K, Karabulut E, Tozum TF (2014). Nitrite and nitrate levels of gingival crevicular fluid and saliva in subjects with gingivitis and chronic periodontitis. JOMR.

[19] Lima SNL, Ribeiro IS, Grisotto MA, Fernandes ES, Hass V, de Jesus Tavarez RR, Pinto SCS, Lima DM, Loguercio AD, Bandeca MC (2018). Evaluation of several clinical parameters after bleaching with hydrogen peroxide at different concentrations: a randomized clinical trial. J Dent.

[20] Bersezio C, Vildósola P, Sáez M, Sánchez F, Vernal R, Oliveira OB, Jorquera G, Basualdo J, Loguercio A, Fernández E (2018). Does the use of a “Walking Bleaching” technique increase bone resorption markers?. Oper Dent.

[21] Bersezio C, Estay J, Sáez M, Sánchez F, Vernal R, Fernández E (2019). Six-month follow-up of the Effect of Nonvital bleaching on IL-1β and RANK-L: a Randomized Clinical Trial. Oper Dent.

[22] Pandis N, Chung B, Scherer RW, Elbourne D, Altman DG (2017). CONSORT 2010 statement: extension checklist for reporting within person randomised trials. BMJ.

[23] Bentz M, Zaouter C, Shi Q, Fahmi H, Moldovan F, Fernandes JC, Benderdour M (2012). Inhibition of inducible nitric oxide synthase prevents lipid peroxidation in osteoarthritic chondrocytes. J Cell Biochem.

[24] CIE Recommendations on Uniform Color Spaces (1977). Color-difference equations, and Metric Color terms. Color Res Appl.

[25] Pérez M, Ghinea M, Rivas R, Yebra MJ, Ionescu A, Paravina AM, Herrera RD (2016). Development of a customized whiteness index for dentistry based on CIELAB color space. Dent Mater.

[26] Gupta G (2013). Gingival crevicular fluid as a periodontal diagnostic indicator-II: inflammatory mediators, host-response modifiers and chair side diagnostic aids. J Med Life.

[27] Alhadlaq AM (2015). Biomarkers of orthodontic tooth Movement in Gingival Crevicular Fluid: a systematic review. J Contemp Dent Pract.

[28] Firat E, Ercan E, Gurgan S, Yucel OO, Cakir FY, Berker E (2011). The effect of bleachıng systems on the gingiva and the levels of IL-1β and IL-10 in gingival crevicular fluid. Oper Dent.

[29] Kapoor P, Monga N, Kharbanda OP, Kapila S, Miglani R, Moganty R (2019). Effect of orthodontic forces on levels of enzymes in gingival crevicular fluid (GCF): a systematic review. Dent Press J Orthod.

[30] Drummond S, Canavarro C, Perinetti G, Teles R, Capelli J (2012). The monitoring of gingival crevicular fluid volume during orthodontic treatment: a longitudinal randomized split-mouth study. Eur J Orthod.

[31] Balladares L, Alegría-Acevedo LF, Montenegro-Arana A, Arana-Gordillo LA, Pulido C, Salazar-Gracez MT, Reis A, Loguercio AD (2019). Effects of pH and application technique of In-office bleaching gels on Hydrogen Peroxide Penetration into the Pulp Chamber. Oper Dent.

[32] Favoreto MW, de Souza Carneiro T, Forville H, Burey A, Simas Dreweck FD, Loguercio AD, Reis A (2023). Use of calcium-containing bioactive desensitizers in dental bleaching: a systematic review and meta-analysis. JADA.

[33] Kwon SR, Wertz PW (2015). Review of the mechanism of tooth whitening. J Esthet Restor Dent.

[34] Pascolutti M, de Oliveira D (2021). A Radical-Free Approach to Teeth whitening. Dent J.

[35] Pinzan-Vercelino CRM, Lima SNL, Pereira F, Gurgel JA, Silva GRD, Freitas KMS. Efficacy of products for bleaching and whitening under orthodontic brackets. Dent Press J Orthod. 2022;27(5). 10.1590/2177-6709.27.5.e2220325.oar PMC9639618 36350943

[36] Paravina RD, Ghinea R, Herrera LJ, Bona AD, Igiel C, Linninger M, Sakai M, Takahashi H, Tashkandi E, Perez MM (2015). Color Difference Thresholds in Dentistry. J Esthet Restor Dent.

[37] Pérez MM, Herrera LJ, Carrillo F, Pecho OE, Dudea D, Gasparik C, Ghinea R, Bona AD (2019). Whiteness difference thresholds in dentistry. Dent Mater.

[38] Claudino D, Ricci WA, Honorio HM, Machry RV, Valandro LF, da Rosa RA, Pereira JR (2021). Spectrophotometric analysis of dental bleaching after bonding and debonding of orthodontic brackets. Saudi Dent J.

[39] Silvestre CF, Rego DB, Arruda CNF, Pires-de-Souza FCP, Regis RR, Negreiros WA, Peixoto RF (2021). Whitening effect of 35% hydrogen peroxide in simulation of tooth with orthodontic bracket. J Esthet Restor Dent.

[40] Sulieman M, Addy M, MacDonald E, Rees JS (2004). The effect of hydrogen peroxide concentration on the outcome of tooth whitening: an in vitro study. J Dent.

[41] Kothari S, Jum’ah AA, Gray AR, Lyons M, Yap K, Brunton M (2020). A randomized clinical trial investigating three vital tooth bleaching protocols and associated efficacy, effectiveness and participants’ satisfaction. J Dent.

[42] Faus-Matoses V, Palau-Martínez I, Amengual-Lorenzo J, Faus-Matoses I, Faus-Llácer VJ (2019). Bleaching in vital teeth: combined treatment vs in-office treatment. J Clin Exp Dent.

[43] Cardenas AFM, Maran BM, Araújo LCR, de Siqueira FSF, Wambier LM, Gonzaga CC, Loguercio AD, Reis A (2019). Are combined bleaching techniques better than their sole application? A systematic review and meta-analysis. Clin Oral Investig.

[44] Levrini L, Paracchini L, Bakaj R, Diaconu A, Cortese S (2020). Dental bleaching during orthodontic treatment with aligners. Int J Esthet Dent.

[45] Sword RJ, Haywood VB (2020). Teeth Bleaching Efficacy during Clear Aligner Orthodontic Treatment. Compend Contin Educ Dent.

